# Impact of Distributing Test Result Reports for Chronic Viral Hepatitis on Awareness of Hepatitis Testing Among Non-Specialist Physicians

**DOI:** 10.3390/medicina61112067

**Published:** 2025-11-20

**Authors:** Jun Itakura, Yutaka Ito, Takashi Suzuki, Yukiko Aoki, Kaori Takamura, Kenji Yajima, Rie Miyahara, Hiroyuki Uetake, Yasuhiro Otomo

**Affiliations:** 1 Department of Gastroenterology and Hepatology, National Hospital Organization Disaster Medical Center, Tokyo 190-0014, Japan; 2 Department of Breast and Gastroenterological Surgery, National Hospital Organization Disaster Medical Center, Tokyo 190-0014, Japan; 3 Department of Nursing, National Hospital Organization Disaster Medical Center, Tokyo 190-0014, Japan; 4 Department of Medical Information, National Hospital Organization Disaster Medical Center, Tokyo 190-0014, Japan; 5 Department of Health Information Management, National Hospital Organization Disaster Medical Center, Tokyo 190-0014, Japan; 6 Department of Critical Care Medicine and Trauma, National Hospital Organization Disaster Medical Center, Tokyo 190-0014, Japan

**Keywords:** HCV, HBV, awareness, distribution, report, non-specialist

## Abstract

*Background and Objectives:* Patients who undergo hepatitis virus testing may remain unaware of their results in the absence of clinician feedback, particularly from non-specialists. To address this issue, we introduced patient-directed test result reports and evaluated their effectiveness in promoting physician responses among non-specialists. *Materials and Methods:* Distribution of hepatitis virus test result reports began on 22 August 2022 at the National Hospital Organization Disaster Medical Center in Japan. The study included all patients who underwent hepatitis testing, excluding those whose tests were ordered by gastroenterologists or screening physicians. The numbers of tests performed and reports distributed were obtained from electronic medical records. *Results:* Between August 2022 and August 2025, 30,700 patients underwent hepatitis B surface antigen (HBsAg) and/or hepatitis C virus antibody (HCV-Ab) testing, and 11,797 individuals received test result reports. In the most recent one-year period (September 2024–August 2025), the report distribution rate was 49.7%, unaffected by test positivity. Compared with the year before report implementation, the referral rates to gastroenterologists increased significantly for both HBsAg-positive (11.4% to 34.5%) and HCV-Ab-positive cases (7.7% to 25.2%; *p* < 0.01). Documentation of test results and confirmation of clinical cure or ongoing treatment by the ordering physician both improved. Cases without physician response decreased markedly (from 61.4% to 23.6% in HBsAg-positive patients and from 59.3% to 24.5% in HCV-Ab-positive patients; *p* < 0.01 for both). *Conclusions:* Distribution of dedicated hepatitis virus test result reports improved awareness among non-specialist physicians and contributed to better management of test-positive patients.

## 1. Introduction

Hepatitis B and C are globally prevalent liver diseases. Both hepatitis B virus (HBV) and hepatitis C virus (HCV) can be transmitted horizontally and vertically. As vertical transmission occurs at birth, individuals infected in this way are often unaware of their infection status. Connecting such asymptomatic carriers of viral hepatitis to appropriate medical care remains a global challenge [1,2,3,4,5,6,7]. A nationwide, government-subsidized screening program for viral hepatitis was conducted in Japan over a five-year period from 2002 to 2007. However, the participation rate was reported to be only approximately 30% of the target population, indicating that a substantial number of infected individuals may remain unaware of their status [8,9,10].

Health checkups represent a good opportunity for viral hepatitis testing, but not all individuals undergo such examinations. The majority of viral hepatitis tests are performed during pregnancy, and prior to various surgical procedures. However, such incidental testing is often not regarded as a matter of major concern, either by patients or healthcare providers. As a result, even if test results are reviewed at the time, they may later be forgotten. Because reminders or notifications regarding tests that have low perceived importance tend to have limited impact, there is a risk that the results of these tests may not be accurately or effectively communicated. In Japan, this issue is regarded as important, and repeated notifications have been issued by the Ministry of Health, Labour and Welfare; nevertheless, their effectiveness has not yet been proven.

If patients with hepatitis virus infections are overlooked, the disease may progress silently, and the risk of developing hepatocellular carcinoma may increase. It is therefore crucial to accurately identify infected individuals and report test results. Moreover, informing patients who test negative is also important, as it can help prevent unnecessary repeat testing in the near future and reduce overall testing costs. In this study, we examined whether issuing written test result reports at the time of hepatitis virus testing could ensure that results are reliably communicated to patients and enhance awareness among healthcare providers who are not specialists in hepatitis.

## 2. Patients and Methods

### 2.1. Pre-Analysis of the Status Trends of Patients with Hepatitis Virus Tests

Prior to the start of the study, we retrospectively reviewed patients for whom HBsAg and HCV-Ab tests had been conducted. Between January 2019 and December 2021, a total of 33,634 hepatitis virus tests were performed. Of these, 28 involved only hepatitis B surface antigen (HBsAg) testing, 133 involved only hepatitis C virus antibody (HCV-Ab) testing, and the remaining 33,473 involved both tests. A total of 2845 tests were ordered by the Department of Gastroenterology and Hepatology and 686 were ordered by the Department of Health Screening; these were excluded from the subsequent analyses. The Department of Critical Care Medicine and Trauma accounted for the largest proportion of tests (10,690 cases, 35.5%), followed by surgical departments (10,237 cases, 34.0%) and internal medicine departments (9149 cases, 30.4%) (Supplementary Table S1). To clarify the clinical settings in which hepatitis virus tests were ordered, in Japan, hepatitis virus testing for surgery or interventional procedures is often performed in outpatient settings. Additionally, emergency patients are at high risk of requiring surgery or interventions in the near future, and testing is frequently conducted immediately upon arrival, concurrently with blood sampling. In this study, such tests were also considered as outpatient tests. We reviewed 905 tests conducted between 1 and 31 January 2021 (Supplementary Table S2). Of these, 810 tests (89.5%) were performed in an outpatient setting, 79 (8.7%) during hospitalization, and 16 (1.8%) as part of routine health checkups.

### 2.2. Preparation and Distribution of the Hepatitis Virus Test Result Reports

A dedicated report form was developed for documenting hepatitis virus test results. On the basis of the preceding considerations, we targeted outpatients and designed the report layout for hand delivery in the outpatient setting. To ensure that no omissions occurred, we decided to provide the report to all individuals tested for hepatitis and determined its content accordingly. The form utilized a checkbox format with three categories: “Suspected hepatitis B,” “Suspected hepatitis C,” and “No evidence of chronic hepatitis virus infection.” In cases marked as “Suspected hepatitis B” or “Suspected hepatitis C,” the report included a message recommending a consultation with a specialist or the patient’s primary care physician. For patients who tested negative for both HBsAg and HCV-Ab, two options were provided: one recommending follow-up testing after an appropriate interval following interventions such as blood transfusions, and another indicating that no further testing was necessary. The content of both versions of the report was reviewed and approved by the Ethics Committee of the National Hospital Organization Disaster Medical Center to ensure compliance with ethical standards.

The distribution of hepatitis virus test result reports commenced on 22 August 2022. Reports were issued to patients with a positive HBsAg or HCV-Ab result and to those who received a negative result. The reports were generated by physicians via the electronic medical records system and directly delivered to the patients in person. While the ordering physician typically issued the report, it was also permissible for other physicians—either within the same department or from another department—to do so. There were no limitations on the number of times a report could be issued for the same test, and physicians were encouraged to proactively distribute these reports. In addition, beginning in September 2024, we started providing the report at the time of admission, as part of the admission documents, to patients who had undergone testing as part of preadmission screening. In this setting, the updated version was used, with the option categories expanded to include “Suspected hepatitis B,” “Suspected hepatitis C,” “No evidence of infection,” and a newly added category, “Test not performed.” This new version was used in parallel with the original outpatient version. Throughout the study period, aside from twice-yearly reminders to distribute reports, no additional lectures, educational sessions, or campaigns regarding viral hepatitis were conducted.

### 2.3. Patients and Data Collection

The study included all patients who underwent hepatitis B surface antigen (HBsAg) testing and hepatitis C virus antibody (HCV-Ab) testing at the National Hospital Organization Disaster Medical Center, Tokyo, Japan, from January to December 2021 and from August 2022 to August 2025. Using the issuance history recorded in electronic medical records, the number of hepatitis virus test result reports distributed each month, along with the ordering status of HBsAg and HCV-Ab tests, was reviewed and summarized. All tests were conducted on the basis of medical necessity, and no additional blood sampling or medication was conducted for research purposes; only medical record data were used for detailed analysis.

All target patients were investigated regarding their hepatitis virus test results (positive or negative), and whether the report was issued for each test was investigated. For patients who tested positive, medical records were reviewed to determine ① whether they were referred to the Department of Gastroenterology and Hepatology during the study period, ② whether further tests of hepatitis virus infection, such as the HBV DNA test or HCV RNA test, were performed by the ordering physician, ③ whether clinical cure (negative HBV DNA/HCV RNA test) or ongoing treatment was confirmed by the ordering physician through medical records, referral letters, or patient inquiries, and ④ whether the hepatitis virus test results were documented in the medical record by the ordering physician. Patients for whom all four of the above criteria were “no” and who did not receive a report were considered to have received no response from healthcare providers regarding their positive test results.

This study was conducted in accordance with the 2013 Declaration of Helsinki, and with the 2018 Declaration of Istanbul, and in accordance with the ethical guidelines for epidemiological research established by the Japanese Ministry of Education, Culture, Sports, Science and Technology and the Ministry of Health, Labour and Welfare. We conducted monthly monitoring of report distribution as part of routine clinical quality assurance. Because the present study required a retrospective review of patient medical records for analysis, ethical approval was obtained post hoc. The study design was approved by the ethics committee of the National Hospital Organization Disaster Medical Center (approval no. 2025-36, approved date: 27 October 2025). Written informed consent was waived due to the retrospective nature of this study, and opt-outs were permitted. 

### 2.4. Statistical Analysis

Statistical analyses were conducted using EZR ver. 1.68 software [11]. The chi-square test and Student’s t-test were employed to assess statistical significance, with a *p*-value of <0.05 considered statistically significant.

## 3. Results

### 3.1. Trends in the Distribution of the Hepatitis Virus Test Result Reports

The distribution of hepatitis virus test result reports commenced on 22 August 2022. Between August 2022 and August 2025, a total of 34,409 hepatitis B surface antigen (HBsAg) tests and 39,908 hepatitis C virus antibody (HCV-Ab) tests were performed in 30,700 patients. After excluding those ordered by the Department of Gastroenterology and Hepatology (2755 cases) or by the Department of Health Screening (623 cases), hepatitis virus testing was conducted in 28,338 cases. Of these, 114 involved HBsAg testing only, 44 involved HCV-Ab testing only, and the remaining 28,280 involved both HBV and HCV testing.

**Figure 1:**
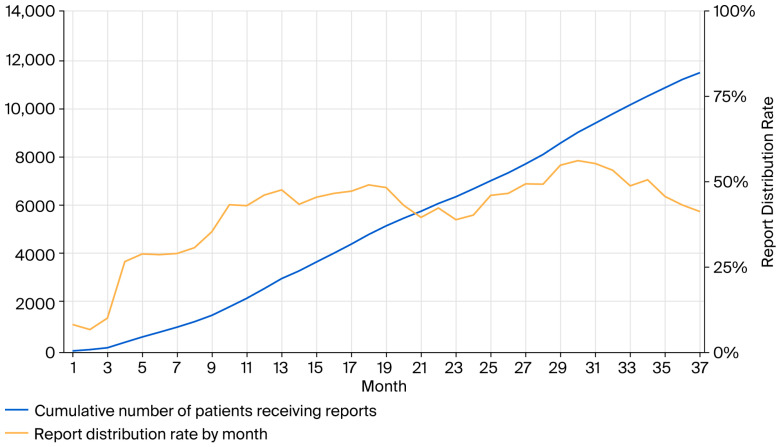
Monthly trends in the cumulative number of cases receiving reports and monthly trends in the report distribution rate. From the initiation of report distribution, the cumulative number of patients receiving reports increased linearly, and the distribution rate also gradually rose. Solid line: Cumulative number of cases receiving reports; Blue line: Number of reports distributed; Orange line: The distribution rate.

Throughout the study period, a total of 13,945 hepatitis virus test result reports were issued. Of these, 405 were issued by the Department of Gastroenterology and Hepatology, and none were issued by the Department of Health Screening; the remaining 13,539 reports were distributed to 11,797 individuals. The cumulative number of patients receiving reports increased linearly, as shown in Figure 1. The distribution rate also gradually increased, reaching approximately 50% over time.

### 3.2. Characteristics of the Pre- and Post-Distribution Groups

To evaluate whether the distribution of hepatitis virus test result reports influenced physician behavior, we reviewed electronic medical records before and after implementation. Specifically, we analyzed cases tested for HBsAg and/or HCV-Ab during two one-year periods, as shown in Table 1: January to December 2021 (pre-distribution; Pre group) and September 2024 to August 2025 (post-distribution; Post group). During the pre-distribution period, 9365 patients underwent hepatitis testing. Of these, 0.68% received only HBsAg testing, 0.31% received only HCV-Ab testing, and the remaining 99.0% received both tests. In contrast, during the post-distribution period, 8915 patients were tested; 0.44% received only HBsAg testing, 0.31% only HCV-Ab testing, and 99.2% both tests. The positivity rate of HBsAg was similar between the groups (0.73% in the Pre group vs. 0.55% in the Post group), as was that of HCV-Ab (1.94% vs. 1.70%, respectively). There were no patients who tested positive for both HBsAg and HCV-Ab in either group, and no positive tests were repeated within the two study periods.

**Figure 2:**
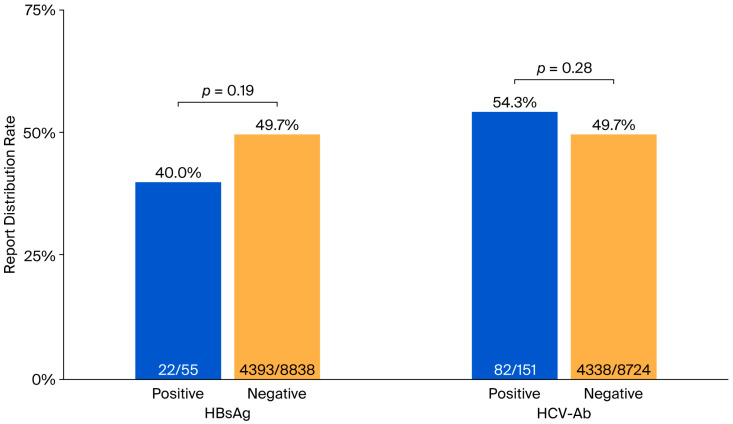
Report distribution rates for positive and negative test results. No significant differences were observed in report distribution rates between positive and negative results for both HBsAg and HCV-Ab tests. There were no patients who tested positive for both HBsAg and HCV-Ab. HBsAg, hepatitis B surface antigen; HCV-Ab, hepatitis C virus antibody.

In the Post group, the percentage of patients who received reports was slightly less than 50% (Table 1). The report distribution rates were 49.7% among patients tested for HBsAg and 49.8% among those tested for HCV-Ab. The positivity rate among patients who received test result reports was comparable to the overall positivity rate of the tested population. We then examined whether the distribution rate differed between patients with positive and negative test results. No significant differences were observed for either test: the distribution rate was 40.0% among HBsAg-positive patients and 49.7% among HBsAg-negative patients (*p* = 0.19), and 54.3% among HCV-Ab-positive patients and 49.7% among HCV-Ab-negative patients (*p* = 0.28), as shown in Figure 2.

### 3.3. Impact of Hepatitis Virus Test Result Report Distribution on Non-Specialist Physicians’ Management of Test-Positive Patients

**Figure 3:**
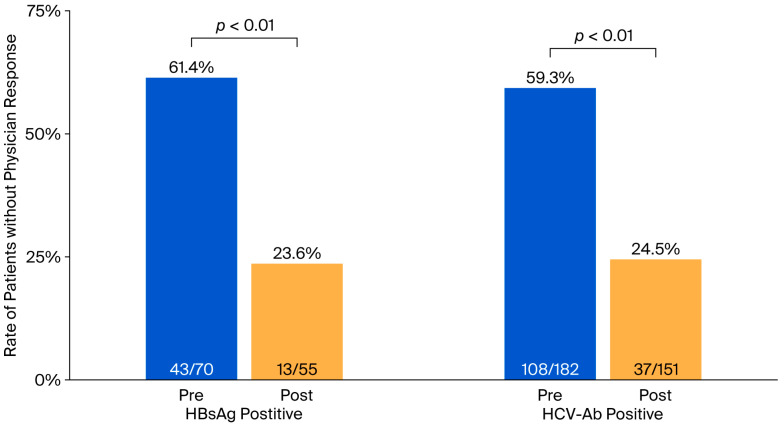
Comparison of the rates of test-positive patients without physician response. The rate without physician response among test-positive patients was significantly lower in the Post group than in the Pre group for both HBsAg and HCV-Ab tests. HBsAg, hepatitis B surface antigen; HCV-Ab, hepatitis C virus antibody.

In the Pre group, among 70 patients who tested positive for HBsAg, 11.4% were referred to a gastroenterologist (Table 2). Similarly, among 182 patients who tested positive for HCV-Ab, only 7.7% were referred. In contrast, in the Post group, 34.5% of 55 HBsAg-positive patients and 25.2% of 151 HCV-Ab-positive patients were referred to gastroenterologists (*p* < 0.01 for both, vs. the Pre group). The rates of further examinations by non-specialist physicians did not differ significantly between the two groups. However, the confirmation rate of clinical cure or ongoing treatment by physicians, assessed through electronic medical records or referral letters, increased markedly following the introduction of the report (from 4.3% to 18.2% among HBsAg-positive patients and from 16.5% to 28.5% among HCV-Ab-positive patients; *p* < 0.05 for both). We also observed a significant increase in the documentation of test results by physicians in medical records (from 34.3% to 61.8% among HBsAg-positive patients and from 25.3% to 39.1% among HCV-Ab-positive patients; *p* < 0.01 for both). Taken together, the proportion of patients without any physician response to positive test results decreased significantly (from 61.4% to 23.6% among HBsAg-positive patients and from 59.3% to 24.5% among HCV-Ab-positive patients; *p* < 0.01 for both; Figure 3 and Table 2).

There were some deceased patients and patients with terminal cancer in both groups. When these cases were excluded from the analysis, similar trends to those described above were observed. In particular, the proportion of patients without physician response decreased by approximately 20% following the implementation of the report distribution (from 57.8% to 22.0% among HBsAg-positive patients and from 57.4% to 18.8% among HCV-Ab-positive patients; *p* < 0.01 for both; Supplementary Table S3).

## 4. Discussion

Chronic hepatitis B has become a more manageable disease owing to the widespread use of nucleos(t)ide analogs, which, while not eradicating the virus, enable effective viral suppression. In contrast, chronic hepatitis C is rapidly being eliminated following the introduction of direct-acting antivirals. The World Health Organization has set a goal to eliminate hepatitis B and C viruses as public health threats by 2030 [12]. However, a major barrier to achieving this goal is the large number of individuals who remain unaware of their infection status. A U.S.-based survey estimated that 0.2% of the population is chronically infected with HBV and that 50.2% of these individuals are unaware of their infection [1]. In Malaysia, only 13% of HBV-infected individuals are aware of their status [2]. Similarly, 50% of individuals with HCV in the United States and 54% in Australia are unaware of their infection [3,4]. In Taiwan, although 41% of people recalled undergoing hepatitis virus testing, only 60% knew their results [5].

The degree to which information—such as hepatitis virus test results—is understood by patients varies depending on the level of interest shown by both the communicator and the recipient. When healthcare providers and patients are both highly engaged, information is more effectively conveyed and more likely to lead to timely diagnosis and treatment. If patients show little interest, even a well-explained message may not be retained. However, disinterested healthcare providers lead to the neglect of critical information, resulting in patients not receiving any relevant information or not understanding the issue even if patients are engaged.

Numerous studies have shown that patient education improves clinical outcomes. For example, a study from the Netherlands reported that clear communication of test results significantly increased specialist referral rates of HBV-positive patients [13]. The importance of appropriate procedures for effective information delivery has also been emphasized in previous studies. In Taiwan, a survey revealed that 12.3% of patients with hepatitis B and 27% of patients with hepatitis C with confirmed infections mistakenly believed that they were not infected [5]. A U.S. study revealed that patients diagnosed at academic hospitals were more likely to receive follow-up care than those diagnosed at community hospitals [14]. More recently, a study in a Korean-American community demonstrated that a hepatitis virus awareness campaign effectively increased specialist referrals for HBV-infected individuals [15]. While large-scale campaigns are widely recognized as being effective at increasing awareness [16], simpler interventions, such as direct notification of positive test results, have also been shown to be effective [17,18].

In contrast, few studies have examined clinician awareness, but this is an essential problem, as described previously. In Uganda, low levels of knowledge about hepatitis B among healthcare providers have been reported [6], and similar concerns have been raised in other regions, such as the Western Black Sea area, emphasizing the need for improved education of medical staff [7]. Furthermore, the effectiveness of standardized care pathways in improving access to specialist care has been demonstrated [19,20]. In our preliminary analysis, more than 90% of hepatitis virus tests were ordered by departments other than gastroenterology or health screening, suggesting that most tests were conducted as part of preoperative or pretreatment assessments for other purposes. In other words, hepatitis testing was not the primary objective for ordering physicians, and their interest in the results was likely limited. To address this, we designed the test result report to be simple to prepare and distribute. Physicians were only required to check a box, while concise instructions for patient follow-up were prefilled, enabling the report to be delivered with minimal explanation. These design features may partly explain the gradual improvement observed in distribution rates, as they lowered the threshold for physicians to provide the reports.

One reason why hepatitis virus test results may be given relatively low priority by physicians is the low frequency of positive findings. A notable feature of our intervention was that test result reports were distributed not only to patients with positive results but also to those with negative results. In this study, the majority of patients who received reports had negative results for both HBsAg and HCV-Ab. Importantly, preparing a report requires the physician to review and confirm each patient’s hepatitis test result, rather than relying on an automated process. This step may have helped draw greater attention to the relatively few positive cases. Although we did not follow up to directly assess the usefulness of distributing reports to patients with negative results, such reports may nevertheless have value in preventing unnecessary repeat testing and in serving as documentary evidence should positive conversion occur in the future.

This study has several limitations. First, it is uncertain whether the observed increase in awareness among non-specialists was attributable solely to the distribution of test result reports. We conducted no additional lectures, educational sessions, or campaigns regarding viral hepatitis during the study period. However, because several physicians rotate annually at our institution, biannual reminders were issued to encourage the distribution of the reports. These reminders may themselves have contributed to heightened awareness; however, as they were exclusively intended to promote report distribution, their effect can reasonably be considered part of the intervention. Second, the distribution method was tailored to the organizational structure of our hospital, which functions as a disaster response facility and includes a relatively large number of physicians in the Department of Critical Care Medicine and Trauma. Therefore, the generalizability of this method to other institutions remains uncertain. Third, in this study, it was not possible to determine how patients responded to healthcare providers upon receiving the test result report. Likewise, we could not assess what actions patients took following receipt of the report. Further studies using methods to clarify patient responses are needed. Fourth, approximately 20–25% of test-positive patients received no response from physicians. This suggests that report distribution alone may be insufficient to ensure comprehensive patient management. Further studies are warranted to explore additional strategies for effective education and communication.

## 5. Conclusions

For patients to acquire knowledge, healthcare providers themselves must possess sufficient knowledge and be committed to communicating it. Thus, a reliable system for information transfer between providers and patients is essential. In this study, the use of a dedicated test result report form enabled accurate communication of hepatitis virus test results to approximately half of the patients and increased awareness among non-specialist physicians. These findings suggest that establishing effective communication methods can enhance non-specialist physicians’ engagement with hepatitis test results, thereby benefiting both patients and healthcare providers. Feedback from patients and healthcare providers would be informative, but if there is little interest, no response can be expected. It is important to foster non-specialist physicians’ interest in hepatitis virus testing without imposing excessive burden, and this study demonstrates a practical approach that may help partially address this issue. Although the report consists of no more than a single sheet of paper, the distribution of test result reports for chronic viral hepatitis may represent a simple yet highly valuable public health tool.

## Figures and Tables

**Table 1:** Background characteristics of non-gastroenterology patients who underwent testing for HBV surface antigen and HCV antibodies (excluding those from the Department of Gastroenterology and Hepatology). All cases were tested by non-specialists in gastroenterology or hepatology.

	Pre * (n = 9365)	Post ** (n = 8923)
Age (years) *^,3^	71 (0–105)	72 (0–103)
Male	55.3%	56.0%
Tested only HBsAg	0.68%	0.51%
Tested only HCV-Ab	0.31%	0.31%
Tested both tests	99.0%	99.2%
Test-positive		
HBsAg-positive	0.73%	0.55%
HCV-Ab-positive	1.94%	1.70%
Report distributed		49.7%
Among tested HBsAg		49.5%
Among tested HCV-Ab		49.8%
Test-positive (among reports distributed)		
HBsAg-positive		0.50%
HCV-Ab-positive		1.86%

HBsAg, hepatitis B surface antigen; HCV-Ab, hepatitis C virus antibody. * Before the distribution of test result reports (January to December 2021); ** after the distribution of test result reports (September 2024 to August 2025); *^,3^ median (min–max).

**Table 2:** Effect of report distribution on non-specialist physicians’ responses. All cases were tested by non-specialists in gastroenterology or hepatology.

	HBsAg-Positive			HCV-Ab-Positive		
	Pre * (n = 70)	Post ** (n = 55)	*p*-Value	Pre * (n = 182)	Post ** (n = 151)	*p*-Value
Male	50.0%	47.3%	0.85	56.0%	58.3%	0.74
Age (years) *^,3^	72 (34–89)	73 (44–93)	0.90	79 (26–98)	77 (41–100)	0.79
Deceased or terminal cancer cases	8.6%	9.1%	1	11.0%	15.2%	0.26
Report distributed	-	40.0%		-	54.3%	
Referred to gastroenterologist *^,4^	11.4%	34.5%	<0.01	7.7%	25.2%	<0.01
Further tests performed *^,5^	7.1%	1.8%	0.23	7.7%	12.6%	0.15
Clinical cure or ongoing treatment confirmed *^,6^	4.3%	18.2%	0.02	16.5%	28.5%	0.01
Results documented in medical records *^,7^	34.3%	61.8%	<0.01	25.3%	39.1%	<0.01
Without physician responses	61.4%	23.6%	<0.01	59.3%	24.5%	<0.01

HBsAg, hepatitis B surface antigen; HCV-Ab, hepatitis C virus antibody; * Before the distribution of test result reports (January to December 2021). ** After the distribution of test result reports (September 2024 to August 2025). *^,3^ Median (min–max). *^,4^ During study period. *^,5^ HBV DNA test or HCV RNA test, performed by the ordering physician. *^,6^ Confirmed by the ordering physician through medical records, referral letters, or patient inquiries. *^,7^ Documented by the ordering physician.

## Data Availability

The data were used during the research but are not publicly available owing to privacy and institutional policy restrictions. Additional information regarding the study, including detailed protocols and statistical analyses, is also available upon reasonable request.

## Supplementary Materials

The following supporting information can be downloaded at https://www.mdpi.com/article/10.3390/medicina61112067/s1: Table S1: Diagnostic test counts by department (excluding the Department of Gastroenterology and Hepatology and the Department of Healthcare Screening) before the distribution of test result reports (January 2019 to December 2021); Table S2: Settings where hepatitis virus tests were performed before the distribution of test result reports (January 2019 to December 2021); Table S3: Effect of report distribution on non-specialist physicians’ responses in surviving cases.

## Abbreviations:

The following abbreviations are used in this manuscript:

HBsAg	hepatitis B virus surface antigen
HCV-Ab	hepatitis C virus antibody
