# Supplementary material for: Impact of Distributing Test Result Reports for Chronic Viral Hepatitis on Awareness of Hepatitis Testing Among Non-Specialist Physicians
Source: Medicina (Kaunas). 2025 Nov 20;61(11):2067. doi: 10.3390/medicina61112067 (PMC12654136; doi:10.3390/medicina61112067)
Supplement: Supplementary file 1 [file medicina-61-02067-s001.zip › medicina-3965483-supplementary.pdf]

Supplementary Table S1. Diagnostic test counts by department (excluding the Department of Gastroenterology and Hepatology and the Department of Healthcare Screening) before the distribution of test result reports (January 2019 to December 2021).

Department	Hepatitis test*	
	(cases)	(%)
Critical Care Medicine and Trauma	10690	35.5
Surgical departments	10237	34.0
Neurosurgery	1459	4.8
Ophthalmology	821	2.7
Oral and maxillofacial surgery	329	1.1
Otorhinolaryngology	59	0.2
Thoracic surgery	322	1.1
Cardiovascular surgery	238	0.8
Breast and gastrointestinal surgery	1662	5.5
Gynecology	604	2.0
Urology	876	2.9
Orthopedic surgery	1863	6.2
Plastic surgery	1158	3.8
Dermatology	833	2.8
Anesthesiology	13	0.04
Internal medicine departments	9149	30.4
Psychiatry	5	0.02
Neurology	807	2.7
Pulmonology	1353	4.5
Cardiology	4464	14.8
Nephrology	686	2.3
Hematology	855	2.8
Diabetes and endocrinology	98	0.3
Collagen disease and rheumatology	633	2.1
General internal medicine	231	0.8
Pediatrics	9	0.03
Radiology	8	0.03
Others	27	0.1
Palliative care and pain management	26	0.08
Nursing	1	0.003

Hepatitis test, hepatitis B surface antigen and/or hepatitis C virus antibody

Supplementary Table S2. Settings where hepatitis virus tests were performed before the distribution of test result reports (January 1 and 31, 2021). All cases were tested by non-specialists in gastroenterology or hepatology.

	Total<br>n = 905	HBsAg<br>n = 899	HCV-Ab<br>n = 894
Outpatient visit	810 (89.5%)	805 (89.0%)	804 (88.8%)
During hospitalization	79 (8.7%)	78 (8.6%)	74 (8.2%)
Health screening	16 (1.8%)	16 (1.8%)	16 (1.8%)

HBsAg, hepatitis B surface antigen; HCV-Ab, hepatitis C virus antibody

Supplementary Table S3. Effect of report distribution on non-specialist physicians' responses in surviving cases. All cases were tested by non-specialists in gastroenterology or hepatology.

	HBsAg-positive			HCV-Ab-positive		
	Pre* (n=64)	Post** (n=50)	p-value	Pre* (n=162)	Post** (n=128)	p-value
Male	72 (34, 89)	73 (44, 93)	0.71	72 (34, 89)	73 (44, 93)	0.74
Age (years)	69.5±12.1	70.1±11.8	0.79	74.5±13.4	74.5±12.8	0.97
Report distributed	-	38.0 %		-	57.0 %	
Referred to gastroenterologist *4	14.1 %	36.0 %	<0.01	6.6 %	28.1 %	<0.01
Further tests performed *5	7.8 %	2.0 %	0.23	6.8 %	13.3 %	0.07
Clinical cure or ongoing treatment confirmed *6	4.7 %	20.0 %	0.02	17.9 %	32.8 %	<0.01
Results documented in medical records *7	37.5 %	68.0 %	<0.01	25.9 %	45.3 %	<0.01
	37.5 %	68.0 %	<0.01	25.9 %	45.3 %	<0.01
Without physician responses	57.8 %	22.0 %	<0.01	57.4 %	18.8 %	<0.01

HBsAg, hepatitis B surface antigen; HCV-Ab, hepatitis C virus antibody

*Before the distribution of test result reports (January to December 2021);

**After the distribution of test result reports (September 2024 to August 2025);

*4 During study period;

*5 HBV DNA test or HCV RNA test, performed by the ordering physician;

*6 Confirmed by the ordering physician through medical records, referral letters, or patient inquiries;

*7 Documented by the ordering physician;
